# Induced Expression of *CYP51a* and *HK1* Genes Associated with Penconazole and Fludioxonil Resistance in the Potato Pathogen *Fusarium oxysporum*

**DOI:** 10.3390/microorganisms11051257

**Published:** 2023-05-10

**Authors:** Yaw A. Akosah, Zarina S. Kostennikova, Marat T. Lutfullin, Guzel F. Lutfullina, Daniel M. Afordoanyi, Semyon G. Vologin, Ayslu M. Mardanova

**Affiliations:** 1 Department of Molecular Pathology, New York University College of Dentistry, New York, NY 10010, USA; 2 Laboratory of Microbial Biotechnology, Institute of Fundamental Medicine and Biology, Kazan Federal University, Kazan 420008, Russialutfullin.marat2012@yandex.ru (M.T.L.);; 3 Department of Agrobiological Research, Tatar Scientific Research Institute of Agricultural Chemistry and Soil Science, FRC Kazan Scientific Center, Russian Academy of Sciences, Kazan 420059, Russia; d.afordoanyi@knc.ru; 4 Laboratory of Molecular Genetics and Microbiology Methods, Kazan Scientific Center of Russian Academy of Sciences, Kazan 420059, Russia; 5 Department of Agrochemical and Biochemical Analysis, Tatar Research Institute of Agriculture, Kazan Scientific Center of Russian Academy of Sciences, Kazan 420059, Russia

**Keywords:** fludioxonil, penconazole, *Fusarium*, *Solanum tuberosum* L., wilt, dry rot, *CYP51*, histidine kinase

## Abstract

Preventing antifungal resistance development and identifying pathogens with high, medium, and low risk of resistance development to a particular fungicide or fungicide class is crucial in the fight against phytopathogens. We characterized the sensitivity of potato wilt-associated *Fusarium oxysporum* isolates to fludioxonil and penconazole and assessed the effect of these fungicides on the expression of fungal sterol-14-α-demethylase (*CYP51a)* and histidine kinase (*HK1*) genes. Penconazole stunted the growth of *F. oxysporum* strains at all concentrations used. While all isolates were susceptible to this fungicide, concentrations of up to 1.0 μg/mL were insufficient to cause a 50% inhibition. At low concentrations (0.63 and 1.25 μg/mL), fludioxonil stimulated growth in *F. oxysporum*. With an increase in the concentration of fludioxonil, only one strain (*F. oxysporum* S95) exhibited moderate sensitivity to the fungicide. Interaction of *F. oxysporum* with penconazole and fludioxonil leads to respective elevated expressions of the *CYP51a* and *HK1* genes, which upsurge with increasing concentration of the fungicides. The data obtained indicate that fludioxonil may no longer be suitable for potato protection and its continuous use could only lead to an increased resistance with time.

## 1. Introduction

Control of the phytopathogens *Fusarium* spp. involves a wide variety of methods, including mechanical, biological, and chemical methods. One of the most common methods is the use of chemical fungicides [1]. Although there are many different fungicides with diverse mechanisms of action, many fungi are reported to be resistant to them [2]. This has raised concerns about the effectiveness of the use of fungicides as a control agent against fungi.

Fungicides of different classes have been certified for use in the fight against plant diseases. Among them, sterol 14α-demethylase inhibitors (DMI), popularly known as azoles are the largest class of compounds with demonstrated efficacy in controlling the disease [3]. They act on 14α-demethylase and CYP51 (a member of the cytochrome P450 family), which is an important regulatory enzyme in the ergosterol biosynthetic pathway. Azole fungicides bind through direct coordination of triazole N-4 or imidazole nitrogen N-3 as the sixth ligand-heme iron [4]. The resulting CYP51-azole complex is catalytically inactive, preventing the demethylation of lanosterol and eburicol, which in turn affects the production of ergosterol—a necessary component in maintaining the fluidity and permeability of fungal cell membranes [5]. Treatment with azoles leads to a decrease in the amount of ergosterol in the cell, which, in combination with the accumulation of 14-demethylated sterols, leads to fungistasis by disrupting the membrane structure and preventing active membrane transport [6]. DMIs top the list of the most common chemical agents used to control plant diseases in the Russian Federation. In recent times, DMIs have been considered to be part of the fungicide groups with inherent medium resistance risk [7]. Although DMIs have been used for many years in agricultural fields, the mechanisms by which fungi develop resistance are still being investigated.

Fludioxonil (FLU) is a non-systemic phenylpyrrole fungicide that has been commercially available in crop protection since the mid-1990s to control a broad spectrum of plant pathogenic fungi in seed, foliar, and postharvest applications [8]. It was initially isolated from *Pseudomonas* sp. and established as a chemical derivative of the natural product pyrrolnitrin [9]. Studies have shown that the drug’s action requires the presence of group III hybrid histidine kinases (HHKs) and the high osmolarity glycerol (HOG) pathway [10,11]. However, its target molecule is still unknown, and its mode of action is incompletely understood. Recently, by heterologously expressing in *Saccharomyces cerevisae* dimorphism-regulating kinase 1 (a class III HHK), it was established that FLU interferes with triosephosphate isomerase (TPI) causing the release of methylglyoxal, which activates the group III HHK and the HOG pathway [12].

The dual-component histidine kinase (HHK) phosphorelay protein complexes are vital constituents of the signal-sensing apparatus of fungi, which facilitate their sensitivity and adaptation to their environment [13]. Filamentous fungi including *Fusarium* spp. possess several HHKs categorized into 11 classes based on their protein sequences. Mutations in class III HHKs have been linked to resistance to certain classes of antifungal agents and are known to sometimes even lead to morphological defects. Class III HKs have been implicated in the dimorphism and virulence of several human pathogens, such as *Aspergillus fumigatus* [14] and *Sporothrix schenckii* [15] and many others. The role of class III HKs in the virulence of plant pathogenic fungi is still less understood. It was established that the silencing of class III HKs strongly declined the virulence in *Botrytis cinerea* [16] and *Colletotrichum lindemuthianum* [17]. In *F. oxysporum*, the two-component group III histidine kinase gene HK1 was found to modulate stress adaptation and virulence. This same gene was reported to control stress response, sclerotia formation, and fungicide resistance in *Sclerotinia sclerotiorum* [18].

Several publications describe the sensitivity mechanisms of plant pathogenic fungi to DMIs and phenylpyrrole fungicides, but only a few focus on their actions in *Fusarium* spp. Exploring the mechanisms of fungicidal resistance in pathogens is valuable for disease management. The resistant basis of DMIs and phenylpyrroles is commonly conferred by (i) mutations in specific respective genes (CYP51, HK, etc.) and/or (ii) increased expression of these genes.

The purpose of this work was to characterize four potato-associated pathogenic isolates of the genus *Fusarium* and to determine the influence of FLU and penconazole (PEN) on the expression levels of *CYP51a* and *HK1* genes in vitro.

## 2. Materials and Methods

Isolates of *Fusarium* spp. were obtained from the root neck of potato plants (*Solanum tuberosum* L. cultivar Zhukovskij rannij) showing symptoms of *Fusarium* wilt. To obtain isolates, root necks of diseased plants were surface sterilized in 0.5% sodium hypochlorite for 2 min with successive thorough rinsing in sterile distilled water and then transferred onto sterile Potato Dextrose Agar (PDA) and Czapek Dox Agar (CDA) supplemented with 50 mg/L chloramphenicol to inhibit bacterial growth [19]. Pure *Fusarium* isolates were transferred onto fresh sterile PDA, with subsequent incubation at 25 ± 2 °C for 7 days under 12 h alternating light (black/white). Obtained fungi were identified as *Fusarium* isolates based on their macro- and microscopic morphology [20].

### 2.1. Molecular Identification of Fungal Isolates

For DNA extraction, aerial mycelia of a 7-day culture were collected and homogenized in liquid nitrogen. DNA was then obtained following Proteinase K and RNase treatment, phenol: chloroform: Isoamyl alcohol extraction, and precipitation with isopropanol [21]. The universal primers ITS1 (5-TCC GTA GGT GAA CCT GCG G-3) and ITS4 (5-TCC TCC GCT TAT TGA TAT GC-3) [22] were used to generate amplicons of the 5.8S regions of the rDNA. Polymerase chain reaction (PCR) was conducted using an MJ Mini Thermal Cycler (BioRad, Hercules, CA, USA) and a PCR ScreenMix-HS kit (Evrogen, Moscow, Russia) according to the manufacturer’s protocol. DNA amplification was performed using the protocol described in [22]. Quality and weight of obtained amplicons were checked on 2% agarose gel electrophoresis run for 60 min under 80 mV, 360 A. PCR products were then purified using PCR Clean Up and Gel Extraction kit (Thermo Fisher Scientific, Waltham, MA, USA) according to the manufacturer’s protocol and sequenced at Evrogen (Moscow, Russia). For species identification, sequences were annotated in the NCBI database using the bioinformatics platform BLAST 2.0 (https://blast.ncbi.nlm.nih.gov/Blast.cgi, accessed on 9 February 2023).

### 2.2. Pathogenicity Test

Three potato cultivars, Zhukovskij rannij (a widely cultivated variety of Russian origin), Red Scarlett (a well-known foreign cultivar, adapted for cultivation in the European part of Russia), and Reggi (a cultivar created recently by the Tatar Research Institute of Agriculture), were assayed for disease susceptibility. In three biological repeats, twenty healthy tuber samples (per cultivar) were tested on each isolate. Tubers of similar size were initially washed, surface sterilized with 70% ethanol, and then wounded to about 4 mm in depth using a sterilized steel pin. A total of 20 μL of *Fusarium* conidia suspended in sterile saline (~10^5^ conidia mL^−1^) was introduced onto each wound, and tubers were left to dry in an aseptic environment for 30 min.

Tubers inoculated with normal sterile saline were used as negative controls. Wounded tubers were wrapped separately in sterile filter papers, placed in sterile labeled paper bags, and incubated in a humid chamber at 25 °C for 14 days. Tubers were then checked after 14 days of incubation for the number of tubers with visible signs of dry rot disease (N_DR_), and the diameters of dry rot (D_DR_) were recorded. Additionally, the duration of tuber health (the number of days tubers remained healthy after inoculation until the appearance of disease symptoms) was recorded for each isolate. Each tuber was then cut across the wound, and the damage was scored based on the proportion of affected tuber tissue. The pathogenicity of each strain was determined by calculating its virulence index [VI] using Equation (1).

VI = [Σ(D_DR_ × N_DR_)/(N_T_ × HD_DR_)] (1)

where N_T_ and HD_DR_ correspond to total number of tested tubers (N_T_ = 10) and the highest D_DR_ recorded in the series.

To confirm Koch’s postulates, tissue samples were collected from both the control and affected tubers, transferred to sterile plates containing PDA, and incubated at 25 ± 2 °C for 4–7 days, and DNA was extracted from the re-isolated pathogenic strains. The BOX- and ERIC-PCR methods, which are used for DNA fingerprinting in bacteria and fungi, were carried out to compare the original and re-isolated pathogenic *Fusarium* spp. [23,24].

### 2.3. In Vitro Test of Isolates on Fungicides

Fungi were first cultured on PDA for 7 days at room temperature. The resulting cultures were used as an inoculum to study their resistance to fungicides. Following sterilization via a 0.22 micron syringe filter, the fungicides FLU (Maxim, Russia, stock concentration = 25 µg/mL) and PEN (Topaz, Russia, stock concentration = 100 µg/mL) were added to the prepared and autoclaved PDA culture media to achieve the following final concentrations (ug/mL): FLU-0.0 (control), 0.63, 1.25, 2.50, and 5.00; PEN-0.0 (control), 0.1, 0.5, 1.0, 1.5, and 2.0. A total of 20 mL of the medium was poured into sterile Petri dishes, and agar plugs with a diameter of about 5 mm were cut out of 7-day-old fungal cultures and placed in the center of PDA plates, supplemented with fungicides. All experiments were carried out in three biological replicates. The cultures were incubated for 6 days in the dark at room temperature, and the diameter of the colonies was measured after 72, 96, 120, and 144 h. Radial mycelia growth inhibition by the fungicide [RGI (%)] was expressed as a percentage and was determined for colonies using the formula: I (%) = [(dc − dt)/(dc − 5)] × 100, where dc is the average colony diameter in the control sample and dt is the average colony diameter in the test sample.

### 2.4. Extraction of Total RNA, cDNA Synthesis, and Gene Expression Study

The expressions of genes in the *Fusarium* isolates with different sensitivities to fungicides were assessed and compared with their respective trends in virulence. *Fusarium* mycelia were homogenized in liquid nitrogen. Total RNA was extracted and purified with an RNA extraction kit (ExtractRNA, Evrogen). The cDNA was generated via reverse transcription reaction with the SuperScript^®^ III CellsDirect cDNA Synthesis Kit (Thermo Fisher Scientific). The obtained cDNA was diluted to a final concentration of 100 ng/μL. Realtime PCR was performed using the real-time PCR kit (qPCRmix-HS SYBR + HighROX, Evrogen). The primers used (Table 1) were designed from the exon nucleotide sequences of the *CYP51* and *HK1* genes using the Primer Blast software [25]. Reactions were carried out on a CFX96 thermocycler (Biorad, USA) in a 20 μL volume containing 10 μL of qPCRmix-HS SYBR + HighROX (Evrogen), 0.25 μM of each primer, and 1 μL (~5 ng/μL) of cDNA. All reactions were conducted in triplicates using the protocol: 95 °C for 3 min, followed by 40 cycles of 95 °C for 10 s and 60 °C for 30 s.

The β-actin gene was employed as a housekeeping gene. ΔCt was calculated using the second derivative maximum (SDM) method following a comparison of the relative expression levels of *CYP51* and *HK1* with B-actin.

### 2.5. Statistical Analysis

The statistical processing of gene expression analysis data, as well as results of the pathogenicity and fungicide tests, was performed using GraphPad Prism version 9.5.1. The statistical results were shown as the mean ± standard error of the mean (SEM). The mean data scores were subjected to the Shapiro–Wilk normality test. Normally distributed data were compared using two-way ANOVA, while data that did not pass the normality test were compared using Friedman’s test, complemented by a *t*-test at the 5% probability level.

## 3. Results

### 3.1. Fungal Isolation and Identification

A total of four fungal isolates belonging to the genus *Fusarium* were obtained from the root necks of sampled wilting potato plants. The predominant appearance of a whitish cottony aerial mycelium with the presence of fusiform and sickle-shaped macroconidia as well as ovoid and curved microconidia indicated the possible affiliation of these fungi to the genus *Fusarium*. Most isolates showed morphology typical to *F. oxysporum* by appearing as delicate, white to pinkish-purple mycelia, often with a bluish-purple tinge.

Amplification of genomic DNA using the ITS primers yielded products, whose electrophoretic bands were observed within a 500–550 bp range. Sequences of the ITS region of the isolates were compared with the National Center for Biotechnology Information (NCBI) database using BLAST 2.0 (http://www.ncbi.nlm.nih.gov/BLAST, accessed on 9 February 2023) database. Sequence data exhibited between 99.7% and 99.9% homology among the tested isolates, and all isolates were identified as *F. oxysporum*.

### 3.2. Pathogenicity Test

**Figure 1:**
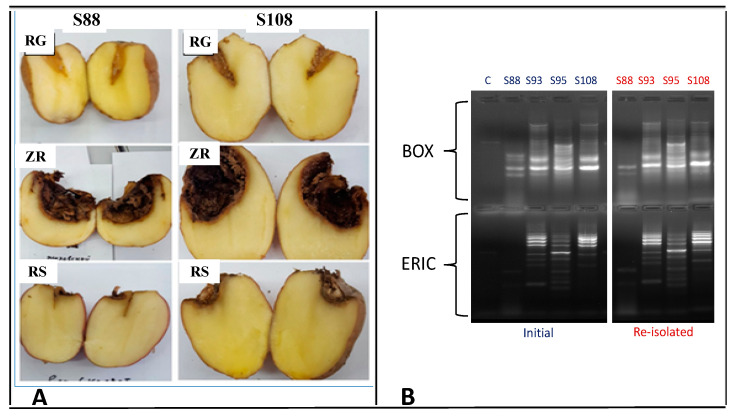
(**A**): Visualization of dry rot in artificially inoculated tubers (Isolates S88 and S108). ZR—Zhukovskij rannij; RG—Reggi; RS—Red Scarlett. Pictures show the different degrees of dry rot (dark brown/black appearance of tissue necrosis in tubers). (**B**): BOX and ERIC−PCR profiles of initial and re-isolated *Fusarium* isolates. ‘C’—control sample, which contained PCR-grade water instead of a DNA template during amplification.

**Figure 2:**
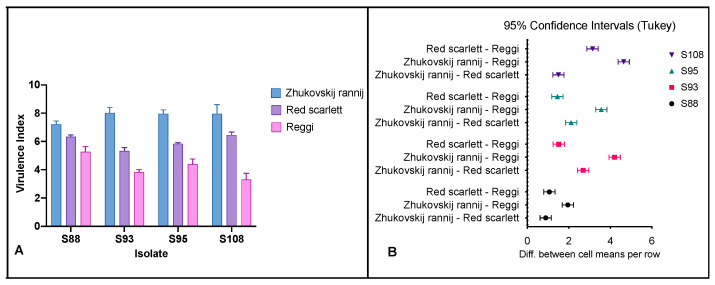
Multiple comparisons of virulence indices (**A**) and 95% confidence interval plots (two-way ANOVA; Tukey) (**B**) of *F. oxysporum* isolates. Virulence indices of strains vary significantly relative to cultivars. The cultivar Zhukovskij rannij is highly susceptible to all four isolates while the lowest virulence is observed in tubers of the Reggi cultivar.

The results of the pathogenicity test showed that all four isolates of *Fusarium* spp. possessed the ability to cause dry rot in at least one of the tested cultivars. Averagely, the onset of dry rot symptoms induced by pathogenic isolates was visible within 3–5 days following inoculation. Wrinkles of the tuber skin over the inoculated area were clearly observed. Incisions made on the 14th day of incubation revealed tissue necrosis with a brownish-black appearance of the internal sections of the tubers (Figure 1A). In some cases, a whitish cotton-like fungal mycelia growth was observed on the affected area. Negative controls did not exhibit any visible signs of dry rot disease. Virulence indices (VI) of strains varied significantly with respect to cultivars (Figure 2). Isolates showed the lowest virulence with regard to tubers of the Reggi cultivar (3.31 ≤ VI ≤ 5.27). The cultivar Zhukovskij rannij appeared to be highly susceptible to all four isolates (7.48 ≤ VI ≤ 8.02). In damaging the Zhukovskij rannij cultivar, the VIs of strains did not significantly vary (*p* > 0.05). However, the highest index of virulence in the Reggi cultivar was registered for *F. oxysporum* S88 (Figure 2).

The repeated isolation of fungal isolates from lesions of infected tubers and their comparative analysis using amplicon bands of the BOX- and ERIC-PCRs confirmed their conformity with Koch’s postulates. Mycelia growth was only observed on plates with tissue samples of affected tubers. Amplicon bands of the BOX- and ERIC-PCRs for the re-isolated fusaria were similar in pattern to that of the original isolates (Figure 1B).

### 3.3. In Vitro Test on Fungicides

**Figure 3:**
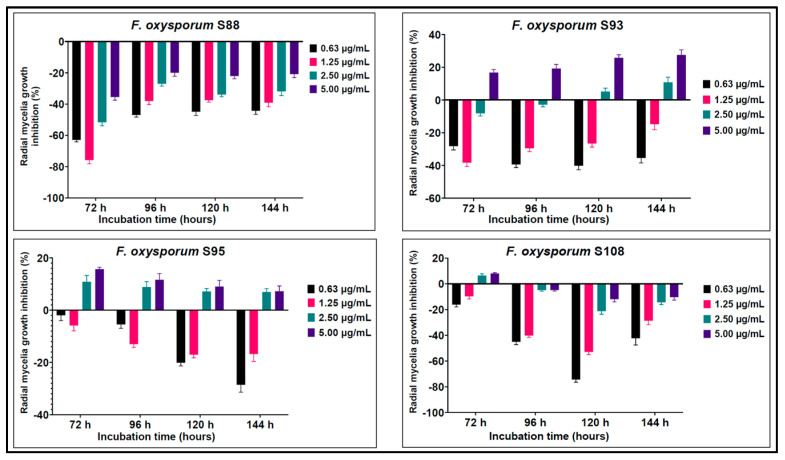
Percent growth inhibition (RGI) of *F. oxysporum* isolates cultured at different concentrations of fludioxonil after 72, 96, 120, and 144 h of incubation. Error bars represent 95% confidence intervals.

The effect of FLU on the *Fusarium* isolates varied significantly. Overall, isolate S88 exhibited no susceptibility to fludioxonil irrespective of the selected concentration or period of incubation. S108 showed a similar pattern with the exception of a slight inhibition at 2.5 and 5.0 µg/mL of FLU within the first 72 h of incubation. However, after 72 h, the strain showed strong resistance to the fungicide. Strains S93 and S95 demonstrated susceptibility to the fludioxonil at concentrations of 2.5 and 5.0 µg/mL. Nonetheless, the fungicide had a positive effect on S93 only after 96 h of incubation. In all cases where the suppression of fungal growth was not observed, the inclusion of FLU led to a significant surge (*p* < 0.01) in mycelia growth in comparison to the control (Figure 3).

**Figure 4:**
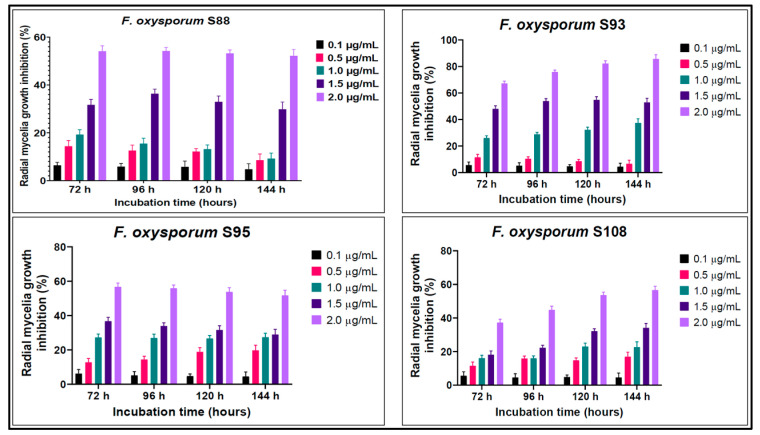
Percent growth inhibition (RGI) of *F. oxysporum* isolates cultured at different concentrations of penconazole after 72, 96, 120, and 144 h of incubation. Error bars represent 95% confidence intervals.

The RGIs of PEN also altered significantly at various concentrations. All the studied *F. oxysporum* strains were susceptible to PEN. Even so, RGI > 50% was only observed at 2.0 µg/mL. At all incubation time periods, the RGIs were directly proportional to the concentration of PEN applied (Figure 4).

**Figure 5:**
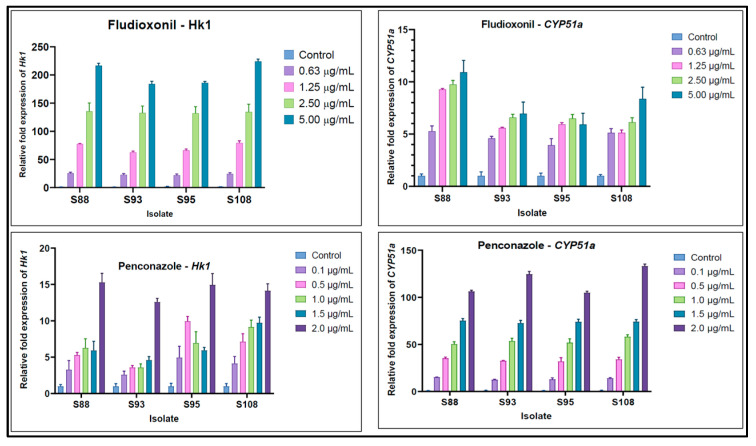
Expression levels of *CYP51* and *HK1* in *F. oxysporum* isolates. Error bars depict a 95% confidence interval (Tukey). To varying degrees, the expression of *CYP51a* and *HK1* is directly affected by fungicide type as well as the concentrations of FLU and PEN.

The expression levels of *CYP51* and *HK1* relative to the β-actin gene (ΔCt) are illustrated in Figure 5. The presence of both fungicides led to an elevation in the relative expression of *CYP51* and *HK1* in all isolates. In addition to the varying degrees of fold increase in expression, fungicide concentration accounted for 86.30–96.65% of the total variance (*p* < 0.001). Expression levels were maximal in isolates at the highest concentrations of PEN and FLU. The expression of *HK1* was relatively higher in the presence of FLU, while *CYP51a* was comparably highly expressed in fungi treated with PEN (Figure 5).

## 4. Discussion

Almost two decades ago, about thirteen species of *Fusarium* had been established to cause wilt and dry rot disease in potatoes [26]. Without a doubt, the number has substantially increased. Some of the primary and, in some cases, the most isolated species are members of the *F. oxysporum* species complex [27], which is possibly due to their high abundance in the soil [28,29]. All isolates being identified as *F. oxysporum* in wilting potato plants was not surprising, since previous studies have reported this species to be the leading cause of wilt in many plants including crops of the *Solanaceae* family [30,31,32,33].

In addition, *F. oxysporum* is numbered among the species responsible for latent *Fusarium* infection in potato tubers [34,35]. Our results tally with the data published in the literature. *F. oxysporum* was confirmed as the most common potato wilt-associated pathogen in the Mid-Volga region of Russia [33], Algeria [36], China [37], and Tunisia [38]. This species was also ranked the leading cause of dry rot in seed potato tubers in Michigan, USA [39]. These results suggest the exceedingly dynamic nature of *Fusarium oxysporum* as a causal agent of potato diseases. The variance in virulence indices (Vis) of isolates relative to the tested cultivars is probably due to the genetic differences in the tested potato cultivars. A previous study has shown that tubers of the cultivar Reggi appear to be more resistant to *Fusarium* isolates in comparison to Zhukovskij rannij and Red Scarlett [40]. In this study, a similar pattern was observed.

Investigating the resistance mechanisms to fungicides in pathogens is crucial for disease control. Such mechanisms are often mediated by mutations in specific genes coupled with increased expression of these genes [2,8]. We performed a comparative antifungal sensitivity analysis of wilt-associated *F. oxysporum* isolates with varying virulence toward potato tubers. As shown in Figure 3, all *F. oxysporum* isolates showed high resistance to FLU at concentrations of 0.63–5.00 μg/mL. Cases of resistance to FLU among *Fusarium* spp. strains have been reported in the literature. For instance, the development of resistance to FLU and other fungicides was observed in the tomato pathogen *F. oxysporum* f. sp. Lycopersici, which reduced the effectiveness of these drugs [41]. Experiments conducted in the United States and United Kingdom also revealed resistance of *F. oxysporum* strains isolated from potato tubers to FLU [39,42,43]. It is known that *F. oxysporum* shows high potential for developing resistance to antifungal drugs that are used repeatedly on tubers due to its genomic plasticity and abundant spore production [44]. Resistant populations of *F. oxysporum* associated with pre- and postharvest losses as well as latent infection of fruits and tubers have also been reported for other members of the *Solanaceae* family [43,45,46].

FLU and other phenylpyrrole derivatives are widely used in the protection of several crops including potatoes [47]. Under laboratory conditions, the effective dose (EC_50_), (the concentration for 50% inhibition of mycelial growth) of FLU in most *Fusarium* species has been reported in the literature to range between 0.002 and 5.0 µg/mL [9,39,48]. The fact that the application of FLU at concentrations of 0.63–1.25 µg/mL resulted in growth stimulation of all *Fusarium* isolates suggests that the studied fungi are probably capable of rapidly incorporating the toxicant into their metabolism as a chemical energy source. This is quite possible, given that *Pseudomonas* spp. (the original source of FLU) and *Fusarium* spp. are soil-abundant microbes [49,50] with root colonizing abilities. Thus, their constant involvement in antagonism and arms race with each other in the near-root zone is inevitable [51]. The rapid development of resistance raises serious concerns about the prospective use of phenylpyrroles such as fludioxonil or their derivatives as antifungal agents in the future.

The other tested fungicide PEN is rarely used in postharvest potato protection though recently recommended [52]. It is however widely used for the preservation of tomato (*Solanum lycopersicum*), which is a close relative of potato [46,53,54]. PEN is also adopted for the preservation of grapes (*Vitis vinifera*) and many other plants that are equally affected by *Fusarium* [55,56,57]. Data on the EC_50_ of penconazole for *Fusarium* are not fully confirmed in the literature, but for other tested mycelial fungi the EC_50_ of PEN has been recorded to be less than 1.0 µg/mL [52,56,58,59]. Based on our results, the EC_50_ dose for the tested isolates was ~1.5–2.0 µg/mL. Palani and Lalithakumari (1999) demonstrated the development of resistance to penconazole in *Venturia inaequalis* using chemical mutagenesis of a wild isolate of a pathogen not previously exposed to penconazole. Later, resistance of *V. inaequalis* to PEN in different apple-growing regions in New Zealand was reported [60]. Moreover, the resistance of *Uncinula necator*, a grapevine pathogen, to PEN was also confirmed [61]. All these data confirm the existence of mechanisms of resistance to penconazole in filamentous fungi.

Recently, a mutation in the *CYP51b* gene and overexpression of *CYP51a* and *CYP51b* were shown to confer multiple resistance to the fungicide prochloraz, a demethylase inhibitor [62]. In addition, the *CYP51* gene family is known to mediate the differential sensitivity of *F. oxysporum* to sterol demethylation inhibitors (voriconazole, prothioconazole, and fluconazole) [63]. Our data on *CYP51a* gene expression suggest that the *Fusarium oxysporum* isolates we tested may also possess similar mechanisms of resistance to penconazole. We performed a comparative analysis of the effects of different concentrations of the fungicides on the expression of *CYP51a* and *HK1* genes in the tested strains of *F. oxysporum*. The *HK1* gene has been identified as a major determinant of FLU resistance in various fungi [10,64]. The expression of *HK1* leads to activation of the High Osmolarity Glycerol (HOG) pathway [12], even though the molecular mechanism of fludioxonil action has been shown to vary from osmotic stress sensing [65]. Being the target of most phenylpyrroles, the inactivation of *HK1* generates *F. oxysporum* resistance to fungicides of the phenylpyrrole and dicarboximide classes [65,66].

The expression levels of *CYP51a* and *HK1* genes varied greatly depending on the gene, strain, and concentration of the fungicide used. While *HK1* expression levels were not significantly increased for penconazole-treated fungi, *CYP51a* expression in the same isolates increased 100–130-fold relative to controls (Figure 5). In contrast, in the presence of 5 µg/mL fludioxonil, there was a strong increase (180–220-fold) in *HK1* gene expression, while *CYP51a* gene expression did not increase more than 6–11-fold.

Thus, penconazole induces *CYP51a* gene overexpression, which is not associated with resistance but appears to be a mechanism of microfungal adaptation to the toxic compound. These data on *CYP51a* overexpression can be used to assess the sensitivity of *F. oxysporum* to penconazole. It has been suggested that an increase in mRNA levels correlates with an increase in cellular levels of *CYP51a*, causing a decrease in pathogen sensitivity to azoles (Price et al., 2015 [6]). Fludioxonil, on the other hand, caused overexpression of the *HK1* gene, and this correlated with the high resistance of isolates to the fungicide, suggesting the involvement of this enzyme in the formation of fungal resistance. HKs have a multifunctional role in transmembrane signaling, and the presence of FLU inhibits their functioning. Thus, the observed overexpression in the *HK1* gene could be a compensatory mechanism for the synthesis of more transmembrane HKs. Therefore, further investigations on the effect of fungicides on the differential expression of various genes are paramount. This will help to devise new control strategies as well as clarify the molecular mechanisms by which phytopathogenic fungi develop resistance to new fungicides.

## 5. Conclusions

A primary agent associated with potato wilt remains *F. oxysporum*, which also shows dry rot-causing potential. At all concentrations, PEN inhibits the growth of the studied *Fusarium* spp. strains. All isolates are susceptible to this fungicide. Notwithstanding, concentrations of up to about 1.0 μg/mL are insufficient to yield a 50% inhibition in the studied strains. FLU does not inhibit fungal growth at low concentrations (0.63 and 1.25 μg/mL) but, on the contrary, stimulates growth in *F. oxysporum*. With an increase in the concentration of fludioxonil, only one strain (*F. oxysporum* S95) exhibits a moderate sensitivity to the fungicide, but a gentle decline in mycelia growth is observed with increasing incubation time. The interaction of *F. oxysporum* with PEN and FLU leads to respective elevated expressions of the *CYP51a* and *HK1* genes, which augments with increasing concentration of the fungicides. This indicates that FLU may no longer be suitable for potato protection against dry rot and its continuous use could only lead to an increased resistance with time. Thus, the progressive resistance to fungicides in phytopathogenic *Fusarium* spp. raises the relevance in the search for prospective alternative environmentally friendly control methods, such as biological and other agricultural techniques.

## Figures and Tables

**Table 1:** Primers used for gene expression analysis.

Gene	Name	Sequence (5′-3′)	Annealing Temperature, °C
Sterol-14-α-demethylase	Fo-CYP51A-dir	AAGGGTAGTGGGGAGACAGTT	60.03
	Fo-CYP51A-rev	GACCAGGCTTCTCAATGTGGA	59.95
Histidine kinase	Fo-HK1-dir	TTTCCTCCTCAAACCTCGCT	58.94
	Fo-HK1-rev	CGCTCTTGTAGCTGCTTCTG	59.00
β-actin	Fo-Act-dir	CTCCCATCAACCCCAAGTCC	60.12
	Fo-Act-rev	AGAAAGTGTAACCGCGCTCA	60.35

## Data Availability

The data obtained in this study are available upon request.

## References

[1] Kaur S., Mukerji K.G. (2004). Potato Diseases and their Management. Fruit Veg. Dis..

[2] Lucas J.A., Hawkins N.J., Fraaije B.A. (2015). The Evolution of Fungicide Resistance. Adv. Appl. Microbiol..

[3] Popiel D., Dawidziuk A., Koczyk G., Mackowiak A., Marcinkowska K. (2017). Multiple Facets of Response to Fungicides–the Influence of Azole Treatment on Expression of Key Mycotoxin Biosynthetic Genes and Candidate Resistance Factors in the Control of Resistant *Fusarium* Strains. Eur. J. Plant Pathol..

[4] Qian H., Duan M., Sun X., Chi M., Zhao Y., Liang W., Du J., Huang J., Li B. (2018). The Binding Mechanism between Azoles and FgCYP51B, Sterol 14α-Demethylase of *Fusarium graminearum*. Pest Manag. Sci..

[5] Emami S., Tavangar P., Keighobadi M. (2017). An Overview of Azoles Targeting Sterol 14α-Demethylase for Antileishmanial Therapy. Eur. J. Med. Chem..

[6] Price C.L., Parker J.E., Warrilow A.G., Kelly D.E., Kelly S.L. (2015). Azole Fungicides—Understanding Resistance Mechanisms in Agricultural Fungal Pathogens. Pest Manag. Sci..

[7] FRAC (2020). Fungal Control Agents Sorted by Cross Resistance Pattern and Mode of Action.

[8] Brandhorst T.T., Klein B.S. (2019). Uncertainty Surrounding the Mechanism and Safety of the Post-Harvest Fungicide Fludioxonil. Food Chem. Toxicol..

[9] Al-Mughrabi K.I. (2010). Biological Control of *Fusarium* Dry Rot and Other Potato Tuber Diseases Using Pseudomonas Fluorescens and Enterobacter Cloacae. Biol. Control.

[10] Lawry S.M., Tebbets B., Kean I., Stewart D., Hetelle J., Klein B.S. (2017). Fludioxonil Induces Drk1, a Fungal Group III Hybrid Histidine Kinase, to Dephosphorylate Its Downstream Target, Ypd1. Antimicrob. Agents Chemother..

[11] Hagiwara D., Asano Y., Marui J., Yoshimi A., Mizuno T., Abe K. (2009). Transcriptional Profiling for Aspergillus Nidulans HogA MAPK Signaling Pathway in Response to Fludioxonil and Osmotic Stress. Fungal Genet. Biol..

[12] Brandhorst T.T., Kean I.R.L., Lawry S.M., Wiesner D.L., Klein B.S. (2019). Phenylpyrrole Fungicides Act on Triosephosphate Isomerase to Induce Methylglyoxal Stress and Alter Hybrid Histidine Kinase Activity. Sci. Rep..

[13] Buschart A., Gremmer K., El-Mowafy M., van den Heuvel J., Mueller P.P., Bilitewski U. (2012). A Novel Functional Assay for Fungal Histidine Kinases Group III Reveals the Role of HAMP Domains for Fungicide Sensitivity. J. Biotechnol..

[14] Spadinger A., Ebel F. (2017). Molecular Characterization of Aspergillus Fumigatus TcsC, a Characteristic Type III Hybrid Histidine Kinase of Filamentous Fungi Harboring Six HAMP Domains. Int. J. Med. Microbiol..

[15] Zhang Z., Hou B., Wu Y.Z., Wang Y., Liu X., Han S. (2018). Two-Component Histidine Kinase DRK1 Is Required for Pathogenesis in *Sporothrix schenckii*. Mol. Med. Rep..

[16] Viaud M., Fillinger S., Liu W., Polepalli J.S., Le Pêcheur P., Kunduru A.R., Leroux P., Legendre L. (2006). A Class III Histidine Kinase Acts as a Novel Virulence Factor in Bortrytis Cinerea. Mol. Plant-Microbe Interact..

[17] Bicalho Nogueira G., dos Santos L.V., de Queiroz C.B., Ribeiro Corrêa T.L., Pedrozo Menicucci R., Soares Bazzolli D.M., de Araújo E.F., de Queiroz M.V. (2019). The Histidine Kinase SlnCl1 of *Colletotrichum lindemuthianum* as a Pathogenicity Factor against *Phaseolus vulgaris* L.. Microbiol. Res..

[18] Duan Y., Ge C., Liu S., Wang J., Zhou M. (2013). A Two-Component Histidine Kinase Shk1 Controls Stress Response, Sclerotial Formation and Fungicide Resistance in *Sclerotinia sclerotiorum*. Mol. Plant Pathol..

[19] Özer G., Mustafa İ., Bayraktar H., Paulitz T., Muminjanov H., Dababat A.A. (2019). First Report of *Fusarium hostae* Causing Crown Rot on Wheat in Azerbaijan. Plant Dis..

[20] Leslie J.F., Summerell B.A. (2008). The Fusarium Laboratory Manual.

[21] Aamir S., Sutar S., Singh S.K., Baghela A. (2015). A Rapid and Efficient Method of Fungal Genomic DNA Extraction, Suitable for PCR Based Molecular Methods. Plant Pathol. Quar..

[22] Dubey S.C., Tripathi A., Singh S.R. (2010). ITS-RFLP Fingerprinting and Molecular Marker for Detection of *Fusarium oxysporum* f. sp.. Ciceris. Folia Microbiol..

[23] Aguilar-Hawod K.G.I., de la Cueva F.M., Cumagun C.J.R. (2020). Genetic Diversity of *Fusarium oxysporum* f. sp. *Cubense* Causing Panama Wilt of Banana in the Philippines. Pathogens.

[24] Mishra R.K., Pandey B.K., Pathak N., Zeeshan M. (2015). BOX-PCR-and ERIC-PCR-Based Genotyping and Phylogenetic Correlation among *Fusarium oxysporum* Isolates Associated with Wilt Disease in *Psidium guajava* L.. Biocatal. Agric. Biotechnol..

[25] Ye J., Coulouris G., Zaretskaya I., Cutcutache I., Rozen S., Madden T.L. (2012). Primer-BLAST: A Tool to Design Target-Specific Primers for Polymerase Chain Reaction. BMC Bioinform..

[26] Cullen D.W., Toth I.K., Pitkin Y., Boonham N., Walsh K., Barker I., Lees A.K. (2005). Use of Quantitative Molecular Diagnostic Assays to Investigate *Fusarium* Dry Rot in Potato Stocks and Soil. Phytopathology.

[27] Stefańczyk E., Sobkowiak S., Brylińska M., Śliwka J. (2016). Diversity of *Fusarium* Spp. Associated with Dry Rot of Potato Tubers in Poland. Eur. J. Plant Pathol..

[28] Garnica M., Nucci M. (2013). Epidemiology of Fusariosis. Curr. Fungal Infect. Rep..

[29] Heltoft P., Brierley J.L., Lees A.K., Sullivan L., Lynott J., Hermansen A. (2016). The Relationship between Soil Inoculum and the Development of *Fusarium* Dry Rot in Potato Cultivars Asterix and Saturna. Eur. J. Plant Pathol..

[30] Trabelsi B.M., Abdallah R.A.B., Ammar N., Kthiri Z., Hamada W. (2016). Bio-Suppression of *Fusarium* Wilt Disease in Potato Using Nonpathogenic Potato-Associated Fungi. J. Plant Pathol. Microbiol..

[31] Gordon T.R. (2017). *Fusarium oxysporum* and the *Fusarium* Wilt Syndrome. Annu. Rev. Phytopathol..

[32] Inami K., Yoshioka-Akiyama C., Morita Y., Yamasaki M., Teraoka T., Arie T. (2012). A Genetic Mechanism for Emergence of Races in *Fusarium oxysporum* f. sp. *Lycopersici*: Inactivation of Avirulence Gene AVR1 by Transposon Insertion. PLoS ONE.

[33] Akosah Y.A., Vologin S.G., Lutfullin M.T., Hadieva G.F., Scyganova N.F., Zamalieva F.F., Mardanova A.M. (2021). *Fusarium oxysporum* Strains from Wilting Potato Plants: Potential Causal Agents of Dry Rot Disease in Potato Tubers. Res. Crops.

[34] Hadieva G., Lutfullin M., Akosah Y., Malova A., Mochalova N., Vologin S., Stasevsky Z., Mardanova A. (2018). Analysis of *Fusarium* Micromycetes, Isolated from Infected Potato Tubers Grown in the Republic of Tatarstan. Dostizheniya Nauk. Tekhniki APK.

[35] Thomsen P.H. (2017). Potato Quality during Storage: Effect of Maturity Level and Ventilation Strategies: Studies on the Storage Disease *Fusarium* Dry Rot. Ph.D. Thesis.

[36] Azil N., Stefańczyk E., Sobkowiak S., Chihat S., Boureghda H., Śliwka J. (2021). Identification and Pathogenicity of *Fusarium* spp. Associated with Tuber Dry Rot and Wilt of Potato in Algeria. Eur. J. Plant Pathol..

[37] Jia R., Kang L., Addrah M.E., Zhang J., Xu L., Zhang Z., Chen W., Liu J., Zhao J. (2023). Potato Wilt Caused by Co-Infection of *Fusarium* spp. and *Verticillium dahliae* in Potato Plants. Eur. J. Plant Pathol..

[38] Ayed F., Daami-Remadi M., Jabnoun-Khiareddine H., El Mahjoub M. (2006). Effect of Potato Cultivars on Incidence of *Fusarium oxysporum* f. sp. *Tuberosi* and Its Transmission to Progeny Tubers. J. Agron..

[39] Gachango E., Hanson L.E., Rojas A., Hao J.J., Kirk W.W. (2012). *Fusarium* spp. Causing Dry Rot of Seed Potato Tubers in Michigan and Their Sensitivity to Fungicides. Plant Dis..

[40] Kostennikova Z., Akosah Y., Mardanova A. (2020). Molecular Identification and Comparative Characterization of *Fusarium* Isolates, Obtained from Potato Plants. E3S Web Conf..

[41] Akram S., Khan S.M., Khan M.F., Khan H.U., Tariq A., Umar U.U.D., Gill A. (2018). Antifungal Activity of Different Systemic Fungicides against *Fusarium oxysporum* f. sp. *Lycopersici* Associated with Tomato Wilt and Emergence of Resistance in the Pathogen. Pak. J. Phytopathol..

[42] Yoshimi A., Kojima K., Takano Y., Tanaka C. (2005). Group III Histidine Kinase Is a Positive Regulator of Hog1-Type Mitogen-Activated Protein Kinase in Filamentous Fungi. Eukaryot. Cell.

[43] Peters J.C., Lees A.K., Cullen D.W., Sullivan L., Stroud G.P., Cunnington A.C. (2008). Characterization of *Fusarium* spp. Responsible for Causing Dry Rot of Potato in Great Britain. Plant Pathol..

[44] van Diepeningen A.D., Brankovics B., Iltes J., van der Lee T.A.J., Waalwijk C. (2015). Diagnosis of *Fusarium* Infections: Approaches to Identification by the Clinical Mycology Laboratory. Curr. Fungal Infect. Rep..

[45] Sandipan P.B., Solanki B.P., Patel N.N., Patel R.L., Verma P.D., Desai H.R. (2016). Efficacy of Different Fungicides Against Dry Rot Pathogen of Potato Caused by *Fusarium* sp. under In Vitro Condition. Cercet. Agron. Mold..

[46] McGovern R.J. (2015). Management of Tomato Diseases Caused by *Fusarium oxysporum*. Crop Prot..

[47] Malyuga A.A., Chulikova N.S., Ilyin M.M., Khalikov S.S. (2022). Fludioxonil-Based Preparations for Protecting Potatoes from Diseases and Their Effectiveness. Russ. Agric. Sci..

[48] Qiu J.B., Yu M.Z., Yin Q., Xu J.H., Shi J.R. (2018). Molecular Characterization, Fitness, and Mycotoxin Production of *Fusarium asiaticum* Strains Resistant to *Fludioxonil*. Plant Dis..

[49] Akosah Y., Lutfullin M., Lutfullina G., Pudova D., Shagimardanova E., Vologin S., Gogoleva N., Stasevski Z., Sharipova M., Mardanova A. (2021). The Potato Rhizoplane Actively Recruits *Fusarium* Taxa during Flowering. Rhizosphere.

[50] Mardanova A., Lutfullin M., Hadieva G., Akosah Y., Pudova D., Kabanov D., Shagimardanova E., Vankov P., Vologin S., Gogoleva N. (2019). Structure and Variation of Root-Associated Microbiomes of Potato Grown in Alfisol. World J. Microbiol. Biotechnol..

[51] Reardon S. (2015). Bacterial Arms Race Revs Up. Nature.

[52] Abdul K.M., Hadia R., Mohammad H.F., Ahsanullah Y., Abdul S.J., Wakil A.S. (2021). Evaluation of in Vitro Antifungal Potential of Several Fungicides against *Alternaria alternata* (Fr.) *Keissler*, the Causal Agent of Potato Brown Spot in Afghanistan. Nov. Res. Microbiol. J..

[53] Salamzadeh J., Shakoori A., Moradi V. (2018). Occurrence of Multiclass Pesticide Residues in Tomato Samples Collected from Different Markets of Iran. J. Environ. Health Sci. Eng..

[54] Polat B., Tiryaki O. (2019). Determination of Some Pesticide Residues in Conventional-Grown and IPM-Grown Tomato by Using QuEChERS Method. J. Environ. Sci. Health B.

[55] Omer A.D., Granett J., Wakeman R.J. (1999). Pathogenicity of *Fusarium oxysporum* on Different Vitis Rootstocks. J. Phytopathol..

[56] Pitt W.M., Sosnowski M.R., Huang R., Qiu Y., Steel C.C., Savocchia S. (2012). Evaluation of Fungicides for the Management of Botryosphaeria Canker of Grapevines. Plant Dis..

[57] Lorenzini M., Zapparoli G. (2015). Occurrence and Infection of *Cladosporium*, *Fusarium*, *Epicoccum* and *Aureobasidium* in Withered Rotten Grapes during Post-Harvest Dehydration. Antonie Leeuwenhoek Int. J. Gen. Mol. Microbiol..

[58] Beresford R.M., Wright P.J., Wood P.N., Park N.M. (2012). Sensitivity of Venturia Inaequalis to Myclobutanil, Penconazole and Dodine in Relation to Fungicide Use in Hawke’s Bay Apple Orchards. N. Z. Plant Prot..

[59] Singh K.P., Singh A., Prasad R.K., Kumar J. (2017). Postharvest Applicaton of Fungicides, Antagonists and Plant Products for Controlling Storage Scab and Rots of Apple Fruits. Indian Phytopathol..

[60] Beresford R.M., Wright P.J., Wood P.N., Park N.M., Larsen N.J., Fisher B.M. (2013). Resistance of *Venturia inaequalis* to Demethylation Inhibitor and Dodine Fungicides in Four New Zealand Apple-Growing Regions. N. Z. Plant Prot..

[61] Hajian Shahri M., Abbaspoor M., Gazanchian A. (2012). Occurrence of Resistance in Grapevine Powdery Mildew (*Erysiphe necator*) to Azoxystrobin+Difenconazole (Ortiva^®^) and Cross Resistance to Penconazole and Hexaconazole in Khorasan Razavi Province. J. Plant Prot..

[62] Zhang Y., Mao C.X., Zhai X.Y., Jamieson P.A., Zhang C.Q. (2021). Mutation in Cyp51b and Overexpression of Cyp51a and Cyp51b Confer Multiple Resistant to DMIs Fungicide Prochloraz in *Fusarium fujikuroi*. Pest Manag. Sci..

[63] Zheng B., Yan L., Liang W., Yang Q. (2019). Paralogous Cyp51s Mediate the Differential Sensitivity of *Fusarium oxysporum* to Sterol Demethylation Inhibitors. Pest Manag. Sci..

[64] Furukawa K., Randhawa A., Kaur H., Mondal A.K., Hohmann S. (2012). Fungal Fludioxonil Sensitivity Is Diminished by a Constitutively Active Form of the Group III Histidine Kinase. FEBS Lett..

[65] Bersching K., Jacob S. (2021). The Molecular Mechanism of Fludioxonil Action Is Different to Osmotic Stress Sensing. J. Fungi.

[66] Rispail N., di Pietro A. (2010). The Two-Component Histidine Kinase Fhk1 Controls Stress Adaptation and Virulence of *Fusarium oxysporum*. Mol. Plant Pathol..

